# Factors Influencing Procurement of Digital Healthcare: A Case Study in Dutch District Nursing

**DOI:** 10.34172/ijhpm.2021.115

**Published:** 2021-08-29

**Authors:** Sander Holterman, Marike Hettinga, Erik Buskens, Maarten Lahr

**Affiliations:** ^1^Health Technology Assessment Unit, Department of Epidemiology, University Medical Centre Groningen, Groningen, The Netherlands.; ^2^Research Group IT Innovations in Healthcare, Windesheim University of Applied Sciences, Zwolle, The Netherlands.; ^3^Department of Operations, Faculty of Economics & Business, University of Groningen, Groningen, The Netherlands.

**Keywords:** Digital Health, District Nursing, Purchasing, Procurement, The Netherlands

## Abstract

**Background:** Digital health is considered a promising solution in keeping health care accessible and affordable. However, implementation is often complex and sustainable funding schemes are lacking. Despite supporting policy, scaling up innovative forms of health care progresses much slower than intended in Dutch national framework agreements. The aim of this study is to identify factors that influence the procurement of digital health particular in district nursing.

**Methods:** A case study approach was used, in which multiple stakeholder perspectives are compared using thematic framework analysis. The case studied was the procurement of digital health in Dutch district nursing. Literature on implementation of digital health, public procurement and payment models was used to build the analytic framework. We analysed fourteen interviews (secondary data), two focus groups organised by the national task force procurement and eight governmental and third-party reports.

**Results:** Five themes emerged from the analysis: 1) rationale 2) provider-payer relationship, 3) resources, 4) evidence, and 5) the payment model. Per theme a number of factors were identified, mostly related to the design and functioning of the Dutch health system and to the implementation process at providers' side.

**Conclusions:** This study identified factors influencing the procurement of digital health in Dutch district nursing. The findings, however, are not unique for digital health, district nursing or the Dutch health system. The results presented will support policy makers, and decision makers to improve procurement of digital health. Investing in better relationships between payer and care provider organisations and professionals is an important next step towards scaling digital health.

## Background

**Key Messages**

** Implications for policy makers**

- Learn from other sectors regarding the procurement of innovation because of the similarities in barriers and possible solutions.
- Stimulate regional cooperation between providers and payer organisations, given the importance of their relationships, using stewardship as guiding principle.
- Support and advocate the use of alternative payment models, these can overcome the flaws and negative incentives of price and volume-based models.

** Implications for the public**

Care providers in district nursing in the Netherlands struggle with the implementation of digital health. The way digital health is being purchased by payer organisations (health insurers and municipalities) causes the most barriers. This is related to the process of purchasing and to the dominant payment model used (fee-for-service). This current practice of purchasing is focused on cost-control and mainly aimed at short-term effects, causing risk-avoidance. Purchasers are confronted with limited capacity and capabilities, and within the dominant ‘market’ model there is low trust between purchasers and providers. The factors identified are however not unique for Dutch district nursing or for digital health. Alternative payment models, such as bundled payment or value-based payment models, might be better suited to purchase digital health. Key for improvement is investing in a better relationship between healthcare providers and payer organisations.

As in many countries, the Dutch government is convinced that digital health contributes to addressing current healthcare challenges, such as the increasing number of chronic patients and multi-morbidity, growing public health concerns, increasing needs for long term care and rising healthcare costs.^1-3^ Notably, digital health has already been advocated in the national health policy since 1996.^4^ Since 2013 its use is being monitored annually,^5^ and periodic policy documents report on the progress and state of digital health.^3^ Over the years, several campaigns and programs were funded to stimulate development, raise awareness, improve digital skills of users and support implementation and scaling of digital health.^6-8^

 Digital health, following the definition of the World Health Organization (WHO), is a broad umbrella term encompassing eHealth (which includes mHealth or Mobile Health) as well as emerging areas, such as the use advanced computing sciences in ‘big data, genomics and artificial intelligence.’^9^ It has the potential to improve access to care, self-management and independent living at home, patient satisfaction, health outcomes and integration and continuity of care.^9-12^ Thus, individual and overall costs associated with healthcare utilisation and travel time of professionals and patients may be reduced.^11^

 In Dutch primary care and elderly care the implementation of digital health lags behind, compared to for example hospital care.^3^ Examples of such digital health applications are automatic medication dispensers and the use of sensors (home automation) to track daily activity and respond to emergency situations.^13^

 As background the Dutch government developed National Framework Agreements with relevant stakeholders to keep healthcare accessible and affordable. Parallel agreements were made for hospital care, primary care, district nursing and mental healthcare.^3,14^ A recurring element in these agreements is the ambition to scale innovative and digital health services, using existing reimbursement possibilities. In this study we focus on the district nursing where the procurement of digital health in district nursing has been slow despite all efforts, according to the stakeholders involved in the National Framework Agreement for district nursing: government authorities (the ministry of Health, Welfare and Sports and the Dutch Healthcare Authority); the representing bodies or umbrella organisations of healthcare providers in elderly care (institutional and home care), health insurers and municipalities; and the Dutch Patient Organisation.

 District nursing is the medical care that is provided in the home-situation of patients and includes for example medication support, wound care, palliative care, case management, and coordination and support of self-management. Digital technology enables the provision of various interventions at patients’ homes to prevent (re)admissions or outpatient clinic visits, for example by telemonitoring patients with chronic conditions or providing specific drug therapies at home. Such interventions are often hospital-led but rely on district nursing for the physical activities.

 Importantly, payment and regulation of district nursing is a combination of three payment schemes within the Dutch health system, namely (1) a competitive insurance for curative care, (2) a locally organised and tax funded social care system and (3) a single payer system for long-term care.^15^ Access is regulated through the district nurse via an assessment and setting an indication for certain types of care and support. Much of the medical care is purchased and reimbursed by health insurers under the first scheme that is regulated through the Health Insurance Act. Additional support of activities in daily living, such as getting (un)dressed, bathing, toileting and feeding is generally purchased and reimbursed by municipalities, ie, the second scheme regulated through the Social Support Act. In case a patient needs long term nursing care in the home situation, the indication is assessed by the Center for Indicating Care and if granted funded through the third national scheme. Purchasing organisations (ie, the health insurers and municipalities) contract care providers for a certain capacity for their population. This process of purchasing is characterised as a care procurement process. A contract details what payer organisations pay for in terms of quality, quantity, types of care, tariffs, place and time ^16^ based on various payment models (such as fee-for-service and bundled payments). A wide range of digital health services technically qualifies for reimbursement either via add-on tariffs or integrated in tariffs for care-as-usual.^17^

 Despite the supporting health policy, programs and reimbursement possibilities, the procurement of digital health in Dutch district nursing lags behind. The aim of this study was to identify the factors that influence the procurement process of digital health in district nursing in the Netherlands. Given the similarities in implementation and procurement processes, the findings of our study may apply to other sectors and countries as well.

## Methods

###  Study Approach

 For this study we used a case study approach, in which we compared multiple stakeholder perspectives in the procurement of digital health in district nursing in the Netherlands.^18^ This way we analysed differences and similarities in perceived factors that affect the uptake of digital health.

###  Data Collection

 We used multiple sources in order to triangulate results: secondary data of interviews, two focus groups and governmental and third-party reports. A flowchart of the data collection and data analysis is presented in Supplementary file 1 – Figure S1.

 First, we performed a non-structured literature search on factors that could influence the procurement process of digital health of care providers by payer organisations. We searched for [contracting, purchasing, procurement, payment models, funding] of [healthcare innovation, integrated care, telehealth, eHealth, digital health] in PubMed and SCOPUS databases and Google Scholar. We included English literature as well as Dutch literature using the corresponding Dutch search terms. This first round of literature search was used to develop a preliminary category system (see paragraph on data analysis) to get a first notion on what categories or factors might play a role and was not intended to be exhaustive.

 Second, we were able to follow and use the work of the task force Contracting of the National Framework Agreement on district nursing. This task force consisted of representatives of stakeholders, including government authorities. The research team (SH, EB and ML) was able to analyse how organisations identified and solved issues in practice and reflect on this from a scientific perspective.

 The data of fourteen semi-structured interviews was received from the task force (ie, secondary data). Interviews were conducted by a not-for-profit non-governmental organisation (NGO) with expertise on implementation and procurement of digital health. Participants were recruited via the coordinator of the task force, based on their participation in the task force or for their specific expertise on the procurement of digital health. The interviews lasted 60 minutes, were taken face-to-face or by telephone and took place between November 2019-January 2020. The interviews were not audio-taped but extensive (textual) interview reports were made. The interview guide started with a brief summary of the ambitions of the National Framework Agreements regarding procurement of digital health. It included open questions on the current issues in procurement of digital health, the perceived barriers and facilitators, the availability and usage of assessment tools, the capacity for procurement (time, people), and the needs for improvement in the procurement process. The interview questions are presented in Supplementary file 1 – Table S1.

 After the interviews, a first focus group was organised by members of the task force in January 2020 with support from the non-for-profit NGO and one of the authors [SH]. Participants of the focus group (n = 17) were representatives of the relevant stakeholders involved and experienced in the care procurement process. Respondents of the interviews were invited as well as several additional experts. An overview of the participants’ type of organisations is presented in Table 1. During this first focus group, the preliminary results of the interviews were presented and discussed in depth. Participants discussed and rated the issues, barriers and facilitators in order to prioritise future actions. Based on the results of the interviews and the first focus group, the taskforcewrote an action plan to overcome the identified barriers and to apply potential facilitators. This action plan was discussed and fine-tuned in a second focus group (n = 16) in February 2020 with all relevant stakeholders and tasks were divided among them. An overview of these action items is presented in Supplementary file 1 – Table S2.

**Table 1:** Overview of Qualitative Data Collected

**Qualitative Data**	**Number of Participants/Documents**
Interviews	
Health insurers (INS)	5
Hospitals (HOS)	2
District nursing providers (DNP)	3
Municipalities (MUN)	2
Other	2
Documents	
Reports/policy documents	8
Focus groups	
Health insurers	6
Long term care providers	7
Governmental organisations (Ministry of Health, Dutch Healthcare Authority)	3
Other	6

 Finally, in a second round of literature search, grey literature was identified using ad hoc online searches, cross-referencing and several documents were received from participating respondents and members of the task force. These documents were used to understand the context and current practice of the procurement of health innovation by payer organisations in the Dutch health system and in district nursing. This included Dutch governmental and third-party evaluation reports.^19-26^

 An overview of qualitative data collected is presented in Table 1.

###  Data Analysis 

 We used Framework Analysis,^27^ a type of thematic analysis, to analyse documents and secondary data from the interviews and focus groups. Thematic analysis consists of qualitative data analysis in which themes are identified, organised, and interpreted. The themes are recurrent and ‘characterise particular perceptions and/or experiences, which the researcher sees as relevant to the research question.’^27^

 Specifically Framework Analysis is suited for explaining barriers using multiple perspectives within the complexity of a health system, to ultimately contribute to the improvement of health services and policy.^27,28^ The systematic approach of the framework analysis, as suggested King and Brooks and Gale et al provides rigour and clarity in the analysis.^27,28^

 Based on the first round of literature search, as described under the data collection section, we used literature on public procurement of innovation,^29^ telehealth service implementation frameworks^30,31^ and payment models^32,33^ to develop a preliminary category system. We followed the recommendation to keep this relatively simple at this stage.^27^ This coding scheme ie, deductive coding, combined with inductive elements, ie, new codes emerging from the data led to a final analytical framework. This way we were able to draw on the reviewed literature while staying open for elements that emerged from the interviews, focus groups discussions and reports.

Box 1 presents an overview of the six steps using this framework analysis.

**Box 1. Steps Taken in the Framework Analysis**

- **Step 1 – familiarisation with the data:** two authors [SH and ML] read the interview reports and documents to get familiarised with the data. Notes were taken and discussed.
- **Step 2 – coding: **two authors [SH and ML] coded four interview reports, one per stakeholder, using the preliminary category scheme. Notes were taken while coding.
- **Step 3 – development of an analytical framework:** the authors who coded the interview reports [SH and ML] discussed the coded sections. Differences in selected sections and used codes were discussed. After agreement, the codes were given a brief description. With this first analytical framework two authors [SH and ML] coded three more interview reports to test, discuss and refine the coding scheme used. The final framework was discussed and agreed on by the research team [SH, EB and ML].
- **Step 4 – applying the analytical framework: **the final analytical framework was applied to the full dataset (14 interview reports, 2 focus groups discussion reports and 7 documents) by the first author [SH] using QDA Miner version 5.0.28. Relevant sections were highlighted and were given a code.
- **Step 5 – chartering data in the framework matrix: **all coded sections from the interview reports, focus group discussion reports and documents were exported from QDA Miner to Microsoft Excel. By providing each data source with metadata, such as type of organisation or type of document, different perspectives could be compared. Categories were separated by tabs, perspectives were highlighted using colours, fragments were scored on relevance and references to the source were noted.
- **Step 6 – interpreting the data: **the matrix was reviewed by two authors [SH and ML], connections were drawn, differences and similarities were highlighted, and ideas or notes were written down. This resulted in a written memo per category that included the underlying codes, a summary of the fragments, illustrative quotes and questions that remained. These memos ultimately were clustered in the five themes, shown in Table 2.

To enhance the credibility and reliability of our study we applied multiple techniques, as suggested by King and Brooks^27^ and Guest et al.^34^ First, in designing the study, we used multiple sources and methods. Second, in the data collection process, we organised participant feedback in the first focus group. Third, in the data analyses process, we applied intercoding comparison, ie, coding independently in steps 2 and 3 (see Box 1), discussing coded sections, the testing and refining of the analytical framework within the research team, and using a coding scheme with descriptions for intercoder reliability. Fourth, we kept an audit trail, ie, tracking successive versions of the thematic framework, noting changes in themes and capture our reasoning in a research memo. Finally, we included quotes in our result section to support our findings.

## Results

###  Sample Description 

 The participants of the interviews and focus groups were working at long-term care organisations providing district nursing (DNP), hospitals (HOS), municipalities (MUN) and health insurers (INS) or their umbrella organisations at various positions (managerial/advisory/operational). None of the participants withdrew upon being informed on the study. An overview of the codes used for the governmental and third-party reports (REP1-8) is presented in Supplementary file 1 – Table S3.

 During the interviews and focus groups, various implementation factors were discussed (eg, the adoption by patients and professionals, skills, General Data Protection Regulation, information technology [IT] interoperability). Although these factors do not directly influence the procurement process or contract details directly, we consider some of them relevant in the results since they are interrelated to the procurement process or payment model.

 Five themes emerged from the framework analysis: (1) rationale; (2) provider-payer relationship; (3) resources; (4) evidence; (5) the payment model (see Table 2). In this section several factors are described for each theme, illustrated with quotes from the interviews and focus groups.

**Table 2:** Description of Themes Resulted From the Framework Analysis

**Theme**	**Description**
1. Rationale	The why of implementing digital health: motive, urgency and vision of healthcare providers at board or clinical level. It makes health organizations redefine their core business and activities.
2. Provider-payer relationship	The relationship between payer organisations and care providers including their history, level of trust, size and market share, bargaining power and access to negotiations.
3. Resources	The resources that are needed to either invest in digital health or to negotiate the contract, including capabilities and capacity of purchasers and sellers, and tools and methods used in procurement.
4. Evidence	Multiple aspects related to the availability, type and importance of evidence. Either to decide on implementation or procurement, or needed for monitoring and evaluation of digital health.
5. Payment model	The characteristics of the payment model that is used or needed, including elements as contract period, bundled health services, included health services and providers, tariffs, incentives, risk-avoidance, financial and quality aims.

####  1. Rationale 

 All participants felt that digital health might contribute to improve the health of citizens and patients, and could improve the quality of care provided. They also considered it as one of the solutions to curb healthcare costs and labour market issues, yet not to be considered an aim in itself. These factors not directly influence the procurement process, yet show the dynamics of implementing digital health before the procurement process even starts.

####  Motive

 Many participants noted that the motives for health organisations to implement digital health vary considerably. Grantsor other governmental funding possibilities are considered motives but with a downside: “I sometimes miss the intrinsic motivation, now things get adopted because of the funding possibilities. Does it then serve its purpose?”(INS1). Or as a health provider said: “There are too many funding possibilities [...]. This distracts from the real problems and pain [....] and does not create the right sense of urgency” (DNP3). Best practises of innovative health services have in common that they were initiated because of capacity-issues in general practitioner (GP) practises and hospitals (including emergency departments), and an ageing population (REP2).

 Providers and payers consider a joint regional vision for reorganising healthcare to be an important precondition. “This requires liberty, capacity and courage. It starts at executive board level in the region” (HOS2). However, in practice a regional vision is considered complex: “Joint accountability is lacking. Feeling the responsibility and act accordingly means sharing both risks and benefits. It now starts only at personal level by people who know each other”(INS4).

####  Taking Initiative

 Health insurers and municipalities differ in their own role and involvement in digital health projects and its implementation. Most do share best practises, while some actively approach innovative digital health suppliers or health organisations and some participate in project organisations. Others are reluctant to do so, since they see it as task for healthcare providers themselves to innovate.

####  Core Business

 The implementation of digital health has had an impact on the activities of organisations in some care pathways and on the role of district nurses and social workers. This sometimes even leads to hard decisions to hold off health services, outsource IT-services or delegate tasks to other domains and therefore reconsider their core business. “A hospital is not an IT-organisation. Data processing [of digital health services] requires a different focus, disruption. Innovative procurement also means: what am I good at and what not? What should I hold off? [...] We should do less and better. [...] We like to keep everything to ourselves. There is resistance to scale down” (HOS1).

####  2. Provider-Payer Relationship

####  Trust

 The contract that is ultimately signed, is the result of a negotiation process that often does not stand on itself. Payers noted that an existing collaboration or partnership is an important facilitator. “A personal relationship, trust and a track record on innovation helps” (INS5). Providers and payers noted this trust and personal relationship is important at executive board level and between purchasers (working at payer organisations) and sellers (working at provider organisations). It requires mutual understanding of stakes, challenges and organisational culture. This trust-based relationship is known to be important in alternative payment models with often long-term contracts. Recent trends in horizontal accountability are thought to support this (REP6).

####  Size 

 Both payers and providers reported that the size of providers matters since implementing digital health requires sufficient resources in terms of investments and expertise on digital health and alternative payment models. Not all providers get the chance to have a conversation on procurement or the possibility to even negotiate, particularly when they are small or have limited market size in a particular region (REP3). Building a personal and trust-based relationship or a track record, stays out of reach for these suppliers.

 By purchasing care from large providers, payers reach scale easier and faster. This is both the case for innovative and regular district nursing care (REP3; REP5). Large providers more often can meet the quality criteria payers demand and in situations where payers have more bargaining power, providers experience the purchasing process as one-directional (REP8).

 Digital health interventions that cover multiple domains and funding schemes (ie, hospital care, district nursing and social support) encounter complex issues in reaching regional let alone national scale. With a lack of standardisation, the contracts often need to be tailor-made. This is challenging since in one municipality citizens have various competing health insurers, and one health insurer has clients in dozens of municipalities. Compared to the hospital sector, the long-term care sector and district nursing in particular, is very fragmented. There are numerous providers, ranging from self-employed to those with thousands of employees and national coverage.

####  Competition

 Regarding competition, participants expressed various views. Although “Considerations regarding competition cause no real barriers [.....] continuously in-fighting for the lowest insurance-premium is at least not supporting” (INS4). Being innovative, enables both providers and payers to outshine. The wish to outshine contradicts the national framework agreements in which health insurers should follow a dominant health insurer in a certain region, regarding contracts made with a provider (REP1).

####  3. Resources

 One of the bottlenecks in the procurement of digital health, is the limited capacity of payers to negotiate and draft the contracts. “The development of innovative contracts takes a lot of time from all partners involved” (INS4). Even when the right expertise is available, operational tasks such as regular yearly procurement gets priority. Health insurers are aware of the time and capacity that is required for providers to implement complex innovations. That is why some health insurers demand “Sufficient skilled project management and professionals, and an implementation strategy” from providers (REP1). Since it takes time before new protocols and changes are embedded in clinical practice, long-term contracts are considered a facilitator (REP1). Primarily municipalities noted the limited budgets available for innovation, due to the cost saving targets they also have.

####  Capabilities

 Procurement of digital health requires knowledge and skills both on digital health and innovative contracts or alternative payment models. For municipalities, the procurement of health and social support services is a new task since 2015. Expertise on digital health is still lacking and “Does not get that much attention, there are so many other challenges” (MUN1). Maturity on digital health varies a lot between providers. “What one provider considers innovation, has been care-as-usual for years for other providers” (INS2). In district nursing the number of innovations is not that big, yet knowledge is shared on a limited scale only.

 Regarding innovative contracting and payment models, most payers and providers express the lack of standardisation in outcome indicators, instruments, contracts and terminology. “Good practises do not get recorded in a standardised way” (INS4). It is impossible to have innovative contracts for each combination of digital health and patient population. [...] This requires a lot of time and capacity and goes beyond a traditional procurement relationship” (INS2), which is “routine purchasing using standard contract elements with process- and structure requirements” because of the high number of care providers (REP8).

 Purchasers (working at payer organisations) do not prioritise getting this knowledge on innovative contracting and they have a short-term focus due to financial targets. They are bound to strict internal procurement guidelines often with a task to realise cost savings. Digital health is disrupting this regular procurement process and is perceived to lead to complexity (REP1). Both providers and payers invest in business intelligence and recruit each other’s staff. “The question is whether this leads to higher quality of care [.....] or to higher overhead costs and a race for more bargaining power” (REP8).

####  Tools

 Providers and payers use various methods and tools to support the procurement process, such as business cases, social return on investment analysis, societal cost-benefit analysis and cost-effectiveness analysis. However, this is not standardised. Some develop their own methods and experience challenges in determining societal benefits and comprehensive costs.

####  4. Evidence

####  Outcomes

 The participants mentioned various patient outcomes digital health can have a positive effect on, such as the ability to stay in control and self-manage, feeling secure and free, quality of life, joy, wellbeing, and living at home as long as possible. Health insurers include these values in the criteria whether to purchase digital health from providers. Or as one health insurer emphasises: “digital health services that did prove their added value, should no longer be optional but implemented by default” (INS4). Also, in addition to this added value, the effort it takes to implement an innovation into clinical practice is an important criterion for payers since it is relevant in scaling opportunities to other regions (REP1).

 Providers question whether the evidence on outcomes is always needed, for example in rather simple forms of digital health or in urgent situations due to shortage of care professionals. This is supported by a recent evaluation that show best practises in innovative care in district nursing led to added value in terms of improvements in processes, although this is not always reflected in the measured outcomes (REP2).

####  Business Case

 The importance of having a positive business case is mentioned by all participants. This is most often expressed from their own organisational perspective, ie, does it lead to savings within their own budget. Whether the societal business case is positive, is apparently of less importance. Although payers set a positive business case as a criterion in their yearly purchase guidelines, in some cases their innovation advisers tend to accept plans as well when the effects are not clear yet on short term, in contrary to their colleagues from the purchasing department. Regarding the societal business case, there seem to be “Challenges in making societal cost benefit analysis. There is a lack of alignment in outcomes, indicators and instruments used by the various stakeholders” (HOS2). Different funding schemes and non-balanced costs and savings makes the collaboration between providers, health insurers and municipalities generally complex.

 Whether the business case is underpinned up front with (scientific) evidence from pilots and/or studies elsewhere, is topic of debate. Some payers indicate the evidence (for example changes in care utilisation and costs) is not always needed initially since they are more interested in the logic or idea behind the innovation while others rely more on data. Sometimes payers question the assumptions some providers used for their business case and whether the expected outcomes are realistic. The presence of trust-based relationship, (as discussed in the theme ‘payer-provider relationship’) seems to be of influence here.

 In general, the evidence base for digital health is unclear due to a variety in methodologies used and results reported, even within their own benchmarks as one health insurer remarked. Providers question whether contributing to this evidence base should be done by individual organisations or by their umbrella organisation.

####  5. Payment Model

 All participants noted various facilitators and barriers that we consider to be related to the payment model of the digital health purchased.

####  Contract Period

 Providers and payers see the number of middle- or long-term contracts grow. In district nursing, it varies per health insurer whether this is only for some preferred, large or efficient providers or aimed to be the standard for all. One health insurer mentioned the benefits: “With these partnerships we can progress on innovation, prevention and transformation. It provides (relative) tranquillity and enables to focus on the content. The issues to deal with do not change, the approach does” (INS4). Long-term contracts are considered to be a means to improve healthcare services and are an important element of strategic healthcare purchasing (REP8).

####  Bundled Care

 Another trend is the rising number of contracts for a bundle of health services from one or multiple providers. This is, however, not without challenges. The current payment schemes, such as diagnose related groups, are usually not facilitating. When payment schemes for health services and social support services need to be bundled, issues occur regarding accounting principles and legislation (REP1, REP5). One health insurer considers bundled payment as a first step towards population management models since “It is impossible to have this kind of [bundled payment] contracts for each combination of digital health application and various patient populations” (INS2).

####  Models

 Various providers consider population-based payment models suited for health services for chronic conditions. In these models, population data and data on health outcomes is required. Although data is available on the severity of health problems and demand for health services within specific population groups in district nursing, the variability in this data turns out too large to build alternative payment models on (REP5).

 In one of the analysed reports (REP1) two interesting alternative payment models were presented. First, the reimbursement of costs directly to the patients for using digital health or self-management applications. This option still has many challenges. Secondly, health insurers would like to be able to purchase digital (health) services directly from suppliers other than health service providers, for example from IT-suppliers.

####  Tariffs

 In district nursing various reimbursement codes and tariffs are possible, either structural or experimental. Digital health can be reimbursed as integrated element of care as usual, which seems the preferred option for most participants, or as an add-on tariff. When new reimbursement codes are created or tariffs are being changed, providers and payers remark the additional effort and time needed to get new contracts arranged. It is challenging to find the right balance between stability and necessary adjustments.

####  Incentives

 Provider and payers mentioned that digital health is hindered by a negative incentive because production is stimulated in a fee-for-service payment model. Preventing hospital (re)admission lowers this production and the allocation of payments among hospital-based specialists (often in group-practice) in non-university clinics is often based on production using a national benchmark. However, as one provider remarked, “Since most hospitals negotiate an overall budget with health insurers, less production in chronic care enables growth in acute and elective care.” Furthermore, incentives for higher production can have positive effects when they lead to concentration of low volume, high complexity care, as long as they are assessed independently (REP4).

 Experiments in district nursing with payments per month or year per patient and bundled payments show positive effects in efficiency, due to stimulation of self-management of patients. However, there is a risk of under-treatment or ‘cherry picking’ only financially interesting patients (REP3; REP6).

 Furthermore, wrong-pocket-issues occur between organisations. And within health insurers “Is still thought and acted in silos and domains” (INS3).

####  Risk

 Providers and payers mention risk-avoiding behaviour from both sides due to financial uncertainty. “Providers do not have an entrepreneurial mind-set, [....] and there is a risk of going bankrupt at the end of the year” (DNP4).

####  Financial and Quality Aims

 Although providers and payers see outcome-based payment models as the way forward, these are not often used due to complexity and the lack of operational instruments and data on quality and patient outcomes (REP1, REP3, REP5, INS1). Using these outcomes for sharing risks and savings is therefore not common.

 Furthermore, the large number of providers and therefore numerous contracts that have to be signed, lead to standardisation and purchasing large volumes and therefore less heterogeneity at providers side. When providers’ results deviate from benchmark data on cost and volume, and that leads to negative financial consequences, they will be less motivated to develop innovative healthcare services (REP8).

## Discussion

 The aim of our study was to identify factors that influence the procurement of digital health in district nursing in the Netherlands. We disentangled the issues and causes of the slow scaling process. Based on the analysis of secondary interview data, focus group data and reports, five themes emerged that reflected the perspectives of both providers (hospitals and long-term care providers) and payers (municipalities and health insurers). The identified factors are often intertwined with the design and functioning of the Dutch health system. Below we discuss how our key findings relate to literature on (healthcare) procurement, payment models and digital health implementation and why this broad approach is necessary to understand the dynamics in health practice.

###  Health System

 Many of the factors we identified across the themes *provider-payer relationship*, *resources* and *payment model* relate to the design and functioning of the Dutch health system and are therefore not limited to district nursing; the managed competition, multiple funding schemes, dominant payment models and procurement processes. Literature on the Dutch health system points out that the insurance based, managed competition in the Netherlands results in different purchaser strategies, governance and influencing styles compared to tax-based or single-payer health systems.^35^ The Dutch health insurers are risk-bearing and accountable.^35^ Dutch healthcare purchasing is mostly transaction oriented, using price and volume-based payment models (such as fee-for-service).^32,36^ Its governance is managerial, financial results are needed on short-term(yearly) to keep insurance fees low, and organisations strive for economies of scale.^16,35^

 The factors related to sustained funding of digital health we identified in the setting of Dutch district nursing are however rather common, also seen from an international perspective. Although reimbursement policies for digital health differ per country or region, they share a lack of stability, set restrictions and requirements and the limited number of services, providers and facilities that are covered.^37-40^ Digital health in theory enables integration of healthcare services over multiple domains, providers and disciplines and therefore likely will continue to develop. As a result, reimbursement policies may never reach the long-term stability and certainty providers are looking for. On short term, as the theme *rationale* showed, grant opportunities might enable health providers to implement digital health. However, it does not create the sense of urgency and joint vision that is needed to reorganise healthcare regionally.

 It is important to realise that many factors within the themes *provider-payer relationship* and *resources* we identified in this case study are not limited to the Dutch context, nor unique for healthcare in general. According to literature on procurement, purchasing and supply management they apply across various sectors; having access to the negotiation process, the advantage of size and market share, having a level-playing field,^29,41^ staff capacity,^42^ and importance of collaborative relationships.^29,43^ In this respect a viable alternative to transaction-oriented purchasing might be value-based procurement. In value-based procurement, resources, such as human capital and capabilities, are important in order to provide high quality products and services. In healthcare however, this value-based approach is not common practice yet due to barriers in resources and relations between providers and payers.^44^

 Our case study shows that limited resources in capacity and capabilities clearly play a role in contracting digital health. Noort et al, in their study on purchasing strategies, noted the high staff turnover of purchasers and a low number of employees with a medical background in Dutch purchasing teams.^35^ As an answer, payers could raise capacity and improve capabilities by training, as Meehan et al also suggest.^44^ However, according to Miller et al,^45^ payers should not focus on training of capabilities, but should instead enable these professionals to use the capacities they already possess, to procure more ‘innovation-friendly.’

 Finally, although sufficient capacity is an important precondition, our results underscore the importance of relational factors in the procurement process. Relationship is a key factor in value-based procurement in general, as Meehan et al point out.^44^ And in such business relationships, trust plays a pivotal role. However, particularly in the Netherlands, trust between providers and insurers is at a low level with higher cost of contracting and monitoring as a result.^46,47^ The public perception is that health insurers do not act in the interest of their enrolees but in their own interest, and interference in the patient-doctor relationship is not accepted.^47^ Yet, improvement is possible by being transparent, providing good customer service and by investing in building relationships with providers.^47^ By investing in more capacity, also smaller providers can get access to the negotiation table and build such a relationship and it enables to jointly work on time intensive development of alternative payment models and long-term contracts that incentivize digital health.

###  Perspective for Change

 Despite all identified barriers and issues, literature and good practises do provide perspective for change, not only for the context of Dutch district nursing, but for (digital) healthcare in general. Namely, recent trends in healthcare show a shift of focus towards total cost of care (multi-year contracts, population-based payments) and value and outcomes of care (value-based payment models, pay-for-performance).^16,31,48-51^ These new payment models are relationship oriented with a long-term horizon and stakeholders appear inclined to invest in cooperation which therefore seem suited for digital health. After all, digital health most often requires investments preceding savings and it takes time to achieve the organisational and cultural changes needed. An important element in these alternative payment models is to steer on quality instead of cost containment. Steering on quality, as health insurers are expected to do, is yet uncommon.^36^ This is due to a lack of transparency and information on quality, legislation hindering selective contracting and consumer insensitivity to steering on quality.^36,52^ In Dutch district nursing a joint quality standard has been developed, yet instruments and a standard set of outcome measures are still lacking and should get more priority.

 Also, instead of redesigning the (governance and financing of) health system to overcome its flaws, theory of stewardship could be more helpful in explaining and improving the triad relationships between payers, providers and patients, as suggested by van Raaij.^16^ The currently dominant view is through the lens of agency theories, in which stakeholders operate in self-interest with maximising utilities as driver. The original design of the Dutch health system intended that providers and payers should exert to act in the best interest of either their patients or enrolees. The principles of stewardship could realign stakeholders with these intentions and guide them in the right direction; taking joint responsibility for keeping healthcare accessible and affordable for the population and other stakeholders. To go from an in-fight for a low premium, transaction orientation and cost-control situation towards transparency, mutual trust between providers and payers, joint investment and risk-sharing requires a step-by-step approach and long-term perspective. The starting point could be some low-hanging fruit ending up with more substantial challenges organisations face. Front-runner organisations demonstrate the potential of alternative payment models within the current health system and good purchasing practises are available, yet these could be better disseminated.

###  Implementation of Digital Health

 Besides factors regarding payment models and the procurement process, challenges remain for care providers to implement digital health as our results show; within the theme *rationale *in particular. Providers struggle to integrate digital health in clinical practice and to demonstrate improvement of the health of citizens and patients and quality of care provided. These results correspond with previous studies on implementation of digital health.^12,39,53,54^ Even though various implementation frameworks might facilitate, implementation remains complex given the numerous barriers at health system, organisational and clinical level.^30,53,55^ On the Global Digital Health Index,^56^ comparing 17 countries on digital health strategies, the Netherlands ranks ninth. They score high (third) on policy activity and strategy (engagement, stakeholder involvement and legal regulation), yet low on the degree of digital health readiness and actual use of key digital health solutions (such as ePriscription and Electronic Health Record exchange). Leading countries are Estonia, Canada, Denmark, Israel and Spain. One of the key observations in that report is that in the Netherlands “due to the enormous diversity in private and public applications and actors, there has been a lack of regulatory consistency and interoperability, and especially of clarity.” Since policy activity and strategy is assumed to precede readiness for digital health and actual use, there is perspective for improvement. Recent efforts to improve interoperability and exchange of health data might close the gap.

 Positive however, is the availability of good practises of digital health interventions in district nursing that do meet the criteria and get procured. These might be the less complicated ones, without bundling of care services, provided by a single provider, for a homogeneous patient population or ‘low-tech-high-impact’-solutions. For example, a telecare alarm for elderly at risk of falling is widely accepted. Sharing these good practises, including implementation guides and standard contracts, is a first step towards scaling. This should be manageable as long as the challenges these interventions have, remain simple as Greenhalgh et al demonstrate in their study on their implementation and evaluation framework.^53^

 One final theme to highlight is the role of evidence. Evidence on the effectiveness of digital health is deemed a prerequisite for the decision on its implementation and procurement. Yet such unambiguous evidence is often lacking. Whether this evidence needs to be ‘scientific’ was topic of debate in the interviews and focus groups. This is also a scholarly discussion. Greenhalgh et al are in favour of viewing evaluation of digital health as a social practice instead of scientific testing.^57^ To have digital health evaluated towards implementation, Mathews et al provide a useful digital health scorecard with technological, clinical, usability and costs components including an assessment whether stakeholders’ requirements are satisfied.^58^

###  Limitations and Future Research

 This study had several limitations. First, we used secondary data from interviews instead of conducting them ourselves. These interviews were already planned by the task force and initiated before our study started. However, we were able to find relevant results where one might question whether primary interview data would have resulted in other themes and factors. What our study added to the results of the interviews and focus groups, is the broader scope using reports and literature on district nursing and from other domains and sectors. This triangulation of data did lead to robust findings.

 Secondly, the interview and focus group data for this study was limited to the setting of district nursing in the Netherlands. Since not all barriers are unique for digital health, healthcare procurement in district nursing or for the Dutch health system, the broader reflection in the discussion makes the findings relevant for other domains, sectors and countries as well.

 Thirdly, shortly after the data was collected and analysed, the coronavirus disease 2019 (COVID-19) pandemic started. COVID-19 had major consequences on elective care, care professionals’ capacity, national health budget, and the financial situation and priorities of organisations. In Dutch district nursing there was a reduction in care utilisation due to various reasons such as lower referrals from hospitals and GP’s.^59^ Some of the identified barriers in this study seemed to vanish; suddenly there was sense of urgency to scale digital health in all sectors and infrastructural investments were made on organisational level and reimbursement criteria were adapted. Although it is not strict a limitation of this study, its timing is particular and societal effects of the pandemic are unprecedented. We believe however that the majority of challenges regarding the procurement of digital health and suitable payment models remain. Fortunately, addressing these challenges is now getting more priority than in previous years.

 Finally, the variety of opinions within one perspective (payer and provider) on certain factors, such as the importance of evidence and the use of business cases, might be the result from different levels of experience or innovativeness of providers and payers. Since we did not study individual cases of successful procurement of digital health, and could not check this mediating effect, further research could reveal the mechanisms and drivers of this success.

 In a future study, the effects of the action plan, as agreed on in the second focus group (summarised in Supplementary file 1, Table S2), could be further evaluated. Given the ongoing COVID-19 pandemic and its consequences, changes to this action agenda might be needed since during the pandemic, digital health has scaled in an unprecedented way, and possibly will remain significant in a post-COVID-19 situation. Still, in these new circumstances the procurement of digital health and payment models need further investigation in which stewardship should be integrated as a guiding principle.

## Conclusion

 Unravelling the factors that affect the uptake of digital health in district nursing shows that these are mostly related to design and functioning of the Dutch health system (including the managed competition, multiple funding schemes and procurement processes) and to the implementation at providers’ side. Our findings are, however, not unique for digital health, district nursing or the Dutch health system. To tackle the challenges, having a broader perspective is important in finding ways to overcome them. Literature on implementation of digital health, healthcare procurement and alternative payment models shows that other domains and sectors did encounter these issues before. This might save resources by learning from others and provides perspective, yet teaches us there are hardly any quick fixes. Specifically for the Dutch setting in which trust between care providers and payer organisations is at a relative low level, investing in a better relationship is key. This is an important next step towards scaling digital health since the recommended value-based procurement and alternative payment models are relationship-oriented models.

## Acknowledgements

 We thank the Task Force Contracting of the National Framework Agreement on district nursing, VitaValley (NGO) and people who cooperated in the interviews and focus groups.

## Ethical issues

 This study did not require the participation of human subjects (patients), nor actual patient data. The secondary interview data and focus group data used, did not include personal data, compliant to the General Data Protection Regulation. The interview data was originally not intended to be collected for scientific research. When this study started, the board of the task force agreed to have the interview reports included as secondary data. Participants were then informed on the study at the beginning of the focus groups and given the opportunity to reject their interview data and withdraw from the study.

 As such, this study was considered “service evaluation” and therefore beyond the scope of the local research ethics committee of the University Medical Centre Groningen.

## Competing interests

 SH reports grants from Noaber Foundation, during the conduct of the study.

## Authors’ contributions

 SH: study concept and design, analysis, drafting the manuscript. MH: critical revision of the manuscript for intellectual content. EB: analysis, critical revision of the manuscript for intellectual content. ML: analysis, critical revision of the manuscript for intellectual content.

## Supplementary files

Supplementary file 1 contains Figure S1 and Tables S1-S3.
