# Supplementary material for: Factors Influencing Procurement of Digital Healthcare: A Case Study in Dutch District Nursing
Source: Int J Health Policy Manag. 2021 Aug 29;11(9):1883–93. doi: 10.34172/ijhpm.2021.115 (PMC9808215; doi:10.34172/ijhpm.2021.115)
Supplement: Supplementary file 1 — contains Figure S1 and Tables S1-S3. [file ijhpm-11-1883-s001.pdf]

Article title: Factors Influencing Procurement of Digital Healthcare: A Case Study in Dutch District Nursing

Journal name: International Journal of Health Policy and Management (IJHPM)

Authors' information: Sander Holterman[1,2]*, Marike Hettinga[2], Erik Buskens[1,3], Maarten Lahr[1]

[1]Health Technology Assessment Unit, Department of Epidemiology, University Medical Centre Groningen, Groningen, The Netherlands.

[2]Research Group IT Innovations in Healthcare, Windesheim University of Applied Sciences, Zwolle, The Netherlands.

[3]Department of Operations, Faculty of Economics & Business, University of Groningen, Groningen, The Netherlands.

(*Corresponding author: s.holterman@umcg.nl)

Supplementary file 1

Figure S1. Flowchart of data collection and analysis.

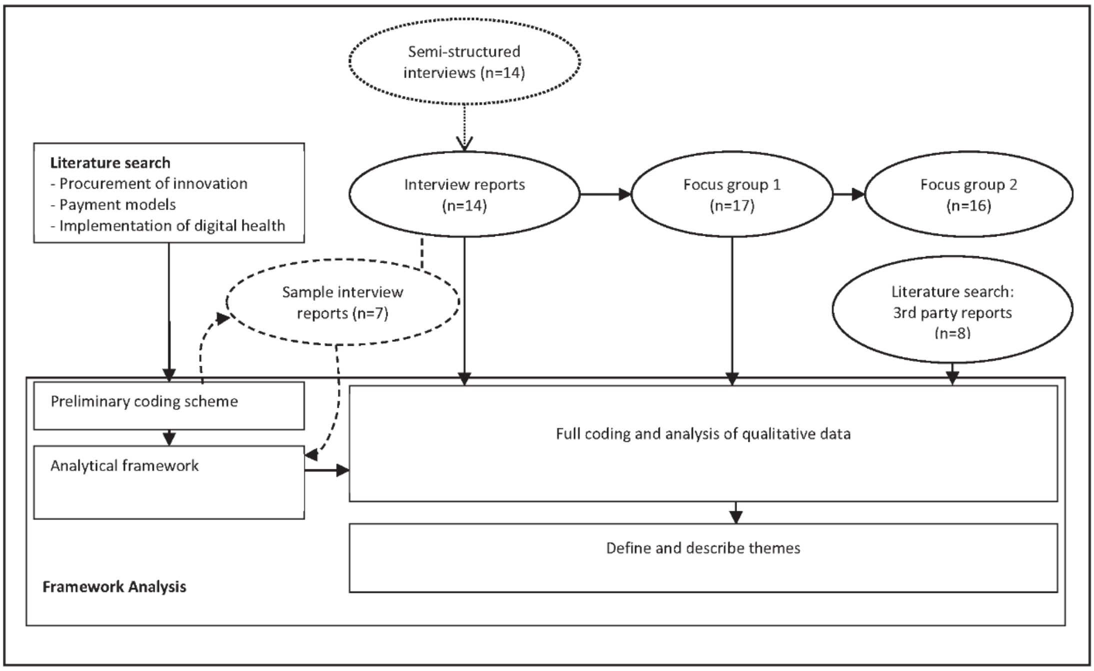

Table S1. Interview questions (translated from Dutch)

1. Is the procurement of innovative and digital care up to speed?
2. What is actually considered to be innovative and digital health? Do stakeholders see the added value of this? And if so, under what conditions?
3. What is the status and what are recent developments within the procurement of such innovative and digital health?
4. Are social costs – benefit analyses made? If yes, how are they performed? And how are business cases being made?
5. Could you elaborate on the needed knowledge, time and capacity for the procurement of innovation? And are there examples and tools to do so?
6. What facilitates and hinders the development and procurement of innovative and digital health?
7. What needs to be done to accelerate this procurement process?
8. What do you consider as your role / responsibility in this?

The interviewers kept asking questions if the first answers were unclear or a deeper understanding was needed, and asked for examples to illustrate specific issues if needed.

Table S2. Actions identified in focus group on Action Agenda

**Activity / goal**
Form leading group of innovative organisations
Develop regional vision on innovation and vary in focus per region
Overview good practises regional cooperation and procurement
Identify good practises of congruent behaviour payers
Facilitate dialogue payers - providers on social innovation
Integrate digital health in education of professionals and students
Include digital health in guidelines and decision support tools
Establish learning community on digital health
Generate overview of (evidence based) good practises digital health
Agree on method for mapping the rationale behind choice of digital health
Establish shared savings principles in contracts
Agree on joint method for measuring societal impact
Develop and share improved model for procurement including use of alternative payment models

Table S3. Reports analysed using the Framework Method

Abbreviation	Title	Author/publisher	Reference
REP1	Procurement of health care innovation	PricewaterhouseCoopers (PwC)	[19]
REP2	Study on scaling regional initiatives Right Care at the Right Place District Nursing / Home Care	ARGO - University of Groningen	[20]
REP3	Monitor Procurement District Nursing 2019	Dutch Healthcare Authority (NZa)	[21]
REP4	Advise on stimulating alignment within theme Wright Care at the Wright Place	Strategies in Regulated Markets (SiRM)	[22]
REP5	Progress Report Development Payment Model District Nursing	Dutch Healthcare Authority (NZa)	[23]
REP6	Advise on Payment Model Physician Care	Dutch Healthcare Authority (NZa)	[24]
REP7	Qualitative and profound supplement on Monitor Procurement District Nursing 2019	Arteria Consulting	[25]
REP8	Care relationship central:Partnership leading for health care purchasing	Council for public health and society (RVS)	[26]
